# Inhibition of USP14 suppresses ferroptosis and inflammation in LPS-induced goat mammary epithelial cells through ubiquitylating the IL-6 protein

**DOI:** 10.1186/s41065-022-00235-y

**Published:** 2022-05-12

**Authors:** Guangqin Zhu, Shaopu Sui, Fengyun Shi, Qinglin Wang

**Affiliations:** Xuzhou City Key Laboratory of Modern AgroBiotechnology, Xuzhou Vocational College of Bioengineering, No. 297 of Sanhuan West Road, Quanshan District, Xuzhou City, 221006 Jiangsu Province China

**Keywords:** Lactoferrin, USP14, Ferroptosis, The NRF signaling pathway, LPS treated goat mammary epithelial cells

## Abstract

**Background:**

Ferroptosis, a novel manner of cell death depended on iron ion, contributed to goat mammary epithelial cell dysfunction. Interleukin-6 (IL-6) is a major pro-inflammatory factor during many inflammation-related diseases including mastitis, and a quite recently identified ferroptosis inducer. This study aims to explore the role of IL-6 in the dysfunction of goat mammary epithelial cells (GMECs) and how the level of IL-6 was regulated.

**Methods:**

Primary GMECs were isolated, cultured and treated with lipopolysaccharide (LPS) alone or together with Ferrostatin-1 (Fer-1), a well-known ferroptosis inhibitor. CCK-8 was used to detect cell viability, ELISA was used to detect TNF-α content, and the levels of ROS, GSH and MDA were analyzed with DCFDA-cell ROS detection kit, GSH assay kit and MDA assay kit, respectively. The iron ion level was measured with an iron assay kit.

**Results:**

The expression level of IL-6 protein in GMECs was up-regulated in response to LPS treatment, and the secretion of TNF-α, the cell oxidative stress level and the Fe^2+^ ion content was robustly increased, which could be reversed by Fer-1 treatment. Knockdown of IL-6 decreased cell oxidative stress level and inhibited ferroptosis in LPS-treated GMECs. Further, ubiquitin experiment and co-immunoprecipitation assay showed that USP14 upregulated IL-6 protein expression by reducing the ubiquitination of IL-6, and overexpression of IL-6 reversed the inhibitory effect of USP14 shRNA on LPS-treated GMECs ferroptosis. The NRF2 inhibitor Brusatol reversed the inhibitory effect of IL-6 shRNA on LPS-treated ferroptosis.

**Conclusion:**

IL-6 protein is deubiquitinated by USP14 and upregulated in LPS-treated GMECs, further promoting ferroptosis and inflammation through the NRF2 signaling pathway.

**Supplementary Information:**

The online version contains supplementary material available at 10.1186/s41065-022-00235-y.

## Introduction

Lactation is an important physiological process of dairy goats. The performance of lactation is closely related to the development of mammary glands. During the growth and development of mammals, mammary epithelial cells (MECs) undergo cell growth, proliferation, differentiation and apoptosis. The MEC is an important model for studying the physiological functions of the mammary gland in vitro and a typical representative of the cells of the lactation system in vivo [1]. It is well known that mastitis changes gene expression and reduces animal performance [2, 3]. Mastitis not only causes huge economic losses to the dairy industry, but also threatens the health of consumers. Escherichia coli (E. coli) is one of the most serious pathogens in epidemiology and the most common bacterium that causes mastitis in dairy cows. E. coli mastitis often shows changes in milk and breasts, such as acute swelling, pain, fever, hard touch in the breast area, watery, bloody or curdled milk, and reduced lactation. At the same time, severely ill animals are often accompanied by systemic symptoms, such as increased body temperature, increased pulse rate, depression and loss of appetite. In the course of mild inflammation, breast swelling, fever, and pain can also be seen in the affected animal, and the milk is abnormal but no blood is seen [4, 5].

Lipopolysaccharide (LPS) is one of the main components of the cell wall of Gram-negative bacteria, which is the basis of pathogenic substances of Gram-negative bacteria. Studies have shown that LPS, as a toxin that induces oxidative damage, has been widely used to establish animal models of inflammation and bacterial infections. LPS activates the NF-κB signaling pathway through toll-like receptor (TLR-4), and promotes the production of downstream inflammatory cytokines including IL-1β, IL-6, TNF-α [6]. It is reported that cell SOD activity was decreased, the level of oxidative stress was increased and cell activity was reduced in LPS treated pheochromocytoma murine adrenoma cell (PC12). This indicated that LPS reduced the activity of PC12 cells by regulating the changes of oxidative stress in the process of cellular inflammation [7]. Recently, LPS was reported to reduce the antioxidant system of weaned piglets, caused oxidative stress in the body, and induced ferroptosis in the jejunal epithelial cells of weaned animals [8].

Ubiquitin specific protease (USP14) is a member of deubiquitylases and belongs to the ubiquitin specific protease family. USP14 could regulate the degradation of target proteins by ubiquitination and facilitate the occurrence and development of inflammation-associated diseases through various signaling pathways [9, 10]. It has been demonstrated that overexpression of USP14 could reduce IκB protein levels and increase LPS induced interleukin (IL)-8 release in lung epithelial cells [11]. Knockdown of USP14 accelerated protein degradation of TNF-α in LPS-stimulated RAW264.7 cells [12]. Furthermore, USP14 has been shown to be involved in ferroptosis, and pharmacological inhibition of USP14 effectively protects neurons from ferroptosis mediated by ferritinophagy [13]. However, whether USP14 plays a role in ferroptosis-mediated dysfunction of goat mammary epithelial cells is unknown.

IL-6 is a pleiotropic cytokine with a wide range of functions, which can regulate the growth and differentiation of a variety of cells, regulate immune response, acute phase response and hematopoietic function, and plays an important role in the body’s anti- infection immune response. IL-6 has been reported dysregulated in a variety of diseases, and its expression imbalance can cause many diseases, mainly manifested by a robust increase in the level at the onset of diseases [14]. IL-6 is rapidly produced in the process of internal trauma, surgery, stress response, infection, and other acute inflammatory reactions. The concentration of IL-6 in surgical patients can predict whether there will be surgical complications [15]. IL-6 also plays an important role in chronic inflammatory reactions, such as atherosclerosis and rheumatoid arthritis [16, 17]. As a main cytokine in response to LPS treatment, IL-6 was also reported to regulate ferroptosis during a few of pathological process, including kidney injury, sepsis, and cartilage injury [18–20]. However, its role in the mastitis was little known.

In this experiment, using LPS stimulated goat mammary epithelial cells (GMECs) as an in vitro inflammation model, we study the effects and mechanism of IL-6 and USP14 on LPS-induced GMEC inflammation and ferroptosis.

## Materials and methods

This study was conducted at Xuzhou Vocational College of Bioengineering and all the animal procedures were approved by the Xuzhou Vocational College of Bioengineering Animal Care and Use Committee (XB19011).

### Cell culture

The primary GMECs were isolated by tissue explant method [21]. After culturing the tissue block for 2–3 weeks, the mixed growth of fibroblasts and mammary epithelial cells were observed under an optical microscope. After 2–3 passages by digestion method, the percentage of GMECs was 99%. The typical “paving stone” or “cobblestone”-like GMECs were observed by light microscope and passaged for subsequent experiments. This method could reduce the damage of trypsin to epithelial cells during the purification process, and obtain mammary epithelial cells with strong proliferation ability. GMECs were cultured in growth medium consisted of 90% DMEM-F12 basal medium (Invitrogen Corp., USA) supplement with 10% fetal bovine serum (FBS, Gibco, USA), 5 mM sodium acetate, 5 mg/L hydrocortisone (Sigma, St. Louis, MO, USA), 5 μg/mL insulin (Sigma, St. Louis, MO, USA), 10 kU/L penicillium/streptomyces (Harbin Pharmaceutical Group, China), 10 ng/mL epidermal growth factor 1 (EGF-1, Gibco), incubated at 37℃ and saturated humidity incubator containing 5% CO_2_. The medium was changed every day. The cells were treated with 1 μg/mL LPS (L4391, Sigma) for 6 h to establish an in vitro inflammation model. When the fusion degree of GMECs reached 70% ~ 80%, CMV-IL-6 expression vector or IL-6 shRNA was added. After 24 h, GMECs were collected, the overexpression and interference efficiency were detected by using RT-qPCR and Western blotting.

### Cell viability assay

The cell suspension (100 μL/well) was inoculated in a 96-well plate and cultured in a 37 °C incubator containing 5% CO_2_. 10 μL of Cell Counting Kit (CCK-8) solution was added to each well, and incubated for 4 h. The absorbance at 450 nm was measured with a microplate reader.

### Enzyme-linked immunosorbent assay (ELISA)

The cell supernatant was collected by centrifuging at 2 000 g for 10 min. The content of TNF-α was detected by using the TNF-α ELISA kit (Zhenke, Shanghai, China). The sample and standard were added to appropriate wells, 50 µL of the antibody cocktail was added to each well and incubated for 60 min at room temperature on a plate shaker set to 400 rpm. After washing the plate 5 times, 100 µL of TMB development solution was added to each well and incubated for 10 min in the dark on a plate shaker set to 400 rpm. Then the plate was washed 5 times, 100 µL of stop solution was added to each well. The plate was shaken on a plate shaker for 1 min, and the absorbance value at 450 nm was recorded.

### Reactive oxygen species (ROS) assay

The ROS level was analyzed by using DCFDA-cell ROS detection kit (ab113851, Abcam, UK). The monolayer GMECs were cultured on a culture plate. After reaching the appropriate cell density, the cells were washed with 1 × buffer, and stained with 20 μM DCFH-DA at 37℃ for 30 min. The cells were washed with 1 × buffer and measured plate immediately on a fluorescence plate reader at Ex/Em = 485/535 nm.

### Glutathione (GSH) assay

The GSH levels were detected by GSH assay kit (Colorimetric, ab239727, Abcam, UK). 100 μL of 5% SSA solution was added into the pelleted cells, vortexed vigorously and kept on ice for 10 min. The supernatant was collected by centrifuging at 12 000 g at 4 ºC for 20 min, and diluted with GSH assay buffer. 10 µL of diluted samples was added to wells of a clear 96-well plate, GSH assay buffer was added to adjust volume to 20 µL/well. 80 µL of the reaction mix was added to standard and sample wells, and the absorbance value at 450 nm was measured by microplate reader.

### Malondialdehyde (MDA) assay

The concentration of MDA in cell lysates was detected by MDA assay kit (thibabituric acid (TBA) method, Jiancheng Institute of Bioengineering, Nanjing, China). The cells were collected and lysed on ice, and the supernatant was taken for detection. The reagents were added to the centrifuge tube according to the kit instructions, mixed evenly, heated in water bath at 95℃ for 40 min, and centrifuged at 4000 rpm for 10 min after cooling in running water. The absorbance value of the supernatant was detected at 532 nm.

### Iron assay

The cell supernatant was collected by centrifugation to detect iron ion content. The iron level was measured by using an iron assay kit (ab83366, Abcam, UK) according to the manufacturer’s instructions. 5 μL of iron reducer was added into the iron standard diluent, 5 μL of iron assay buffer was added into the cell supernatant diluent. The microplate was incubated at 37 °C for 30 min, 100 μL of iron probe was added and incubated at 37 °C for 60 min in the dark. The absorbance value was measured at 593 nm, a standard curve was drawn, and the content of Fe^2+^ in the sample was calculated.

### Ubiquitin experiment

CMV-USP14 vector or USP14 shRNA was transfected into GMECs together with Ub-HA vector, and MG132 was added to inhibit the degradation of proteasome. Cells were lysed in ice and the lysate were incubated with protein A/G agarose beads at 4 ℃ for 3 h, and subsequently incubated with the corresponding antibody overnight at 4 ℃. After washing the agarose beads with PBS, the protein was eluted by boiling in the loading buffer. The protein expression level was detected by Western blotting.

### Co-immunoprecipitation (co-IP) assay

To detect the interaction between IL-6 and USP14, CMV-USP14 plasmid and empty plasmid was transfected into GMECs. The cells were lysed with IP lysis buffer for 15 min on ice, the supernatant was collected by centrifugation at 13,000 g for 10 min. The lysate was incubated with 100 μL magnetic beads binding with USP14 antibody at 4℃ overnight. The agarose beads-antigen–antibody complex was collected by centrifugation briefly at 14,000 rpm for 5 s, washed 3 times with pre-cooled PBS, suspended with 60 μL 2 × loading buffer, and boiled for 5 min to free antigen, antibody, beads. The supernatant was collected by centrifugation for electrophoresis.

### Western blotting

The cells were collected by centrifugation, and lysed with PIPA lysis buffer containing protease inhibitors on ice for 20 min. The supernatant was collected, and the protein concentration was measured by BCA method. The protein was denatured by adding a corresponding volume of 4 × loading buffer and boiling water bath for 10 min. Sodium dodecyl sulfate polyacrylamide gel electrophoresis (SDS-PAGE) was performed to separate the protein. The target protein bands were transfered to the PVDF membranes, blocked in skim milk at room temperature for 2 h, and incubated with the corresponding primary antibody overnight at 4 °C. Next day, the membranes were washed 5 times with TBST, incubated with the secondary antibody at room temperature for 1.5 h. After washing with TBST, the bands were visualized by using the ECL reagent, and quantified by using ImageJ software.

### Transmission electron microscope (TEM)

GMECs were washed with PBS and collected by centrifugation, then fixed by 2.5% glutaraldehyde at room temperature for 1 h followed by further fixation at 4 ℃ overnight. After post-fixation by 1% osmium tetroxide for 1 h, cells were dehydrated with graded ethanol solutions (30, 50, 70, 80, 95, and 100%) and sodium sulfate anhydrous treated acetone. After infiltrating with a 1:1 mixture of acetone and EPON resin for 1.5 h, 100% EPON resin overnight, cells were then embedded in epoxy resin and cured at 60 ℃ for 48 h. Ultra-thin sections of 70 nm were cut by a Leica ultramicrotome. The characteristic changes of ferroptosis were observed under TEM (FEI Tecnai G2 Spirit Twin, ThermoFisher Scientific, USA) after double staining with uranyl acetate and lead nitrate.

### Statistical analysis

The results were expressed as means ± standard deviation of triplicate determinations. The data were analyzed by using SPSS version 23.0 software. *t* test was used to analyze the data of two groups, and the data between multiple groups were compared by One-Way analysis of variance (ANOVA). *P* < 0.05 was considered to indicate statistically significant differences.

## Results

### LPS stimulates ferroptosis in GMECs

**Fig. 1:**
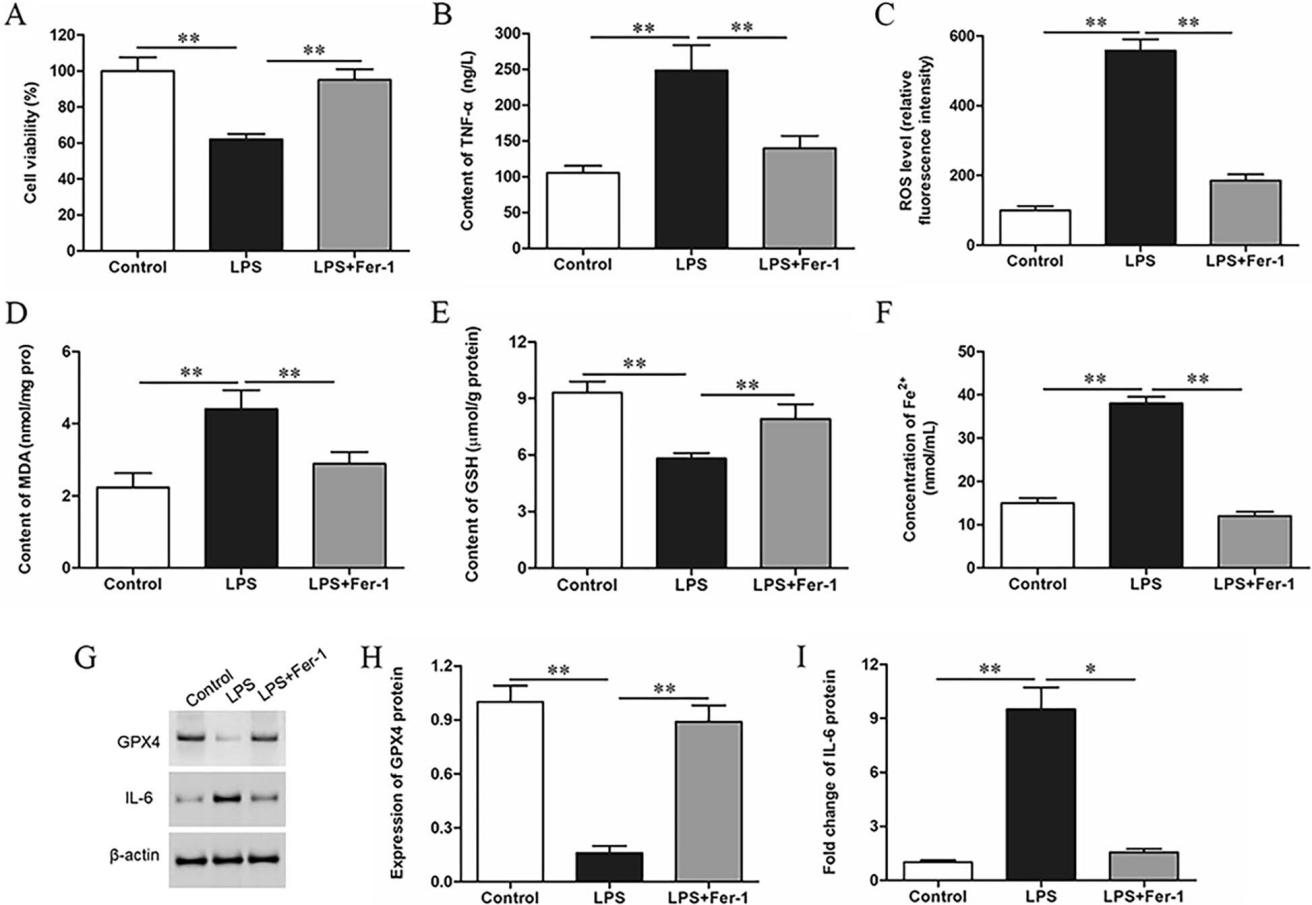
LPS stimulates ferroptosis of GMECs. GMECs were divided into 3 groups, one group was treated with LPS, one group was treated with LPS and Fer-1, and the control group was not treated at all. **A** Cell viability of GMECs was detected by CCK-8 assay. **B** The secretion of TNF-α was detected by ELISA. **C** The content of ROS was analyzed with DCFDA-cell ROS detection kit. **D** MDA content was detected by MDA assay kit. **E** The GSH levels were detected by GSH assay kit. **F** The concentration of Fe^2+^ was measured by using an iron assay kit. **G-I** The expression level of GPX4 and IL-6 was detected by Western blotting and the grayscale was analyzed by using ImageJ software

The cell viability of GMECs was decreased significantly after LPS treatment (Fig. 1A), the secretion of inflammatory factor TNF-α was increased (Fig. 1B), the content of ROS and MDA was increased (Fig. 1C, D), the content of GSH was decreased (Fig. 1E), it meant that the cellular oxidative stress level was increased. In LPS treated cells, the concentration of Fe^2+^ was increased (Fig. 1F), the expression level of GPX4 was decreased, and the expression level of IL-6 was up-regulated (Fig. 1G-I). Ferrostatin-1 (Fer-1), a ferroptosis inhibitor, reduced the oxidative stress level and inflammatory response of cells (Fig. 1B-E), down-regulated Fe^2+^ concentration (Fig. 1F), up-regulated the expression of GPX4 and cell survival rate (Fig. 1A, G-I). These results indicated that ferroptosis played an important role in LPS-treated GMECs.

### Knockdown of IL-6 inhibits ferroptosis in LPS treated GMECs

**Fig. 2:**
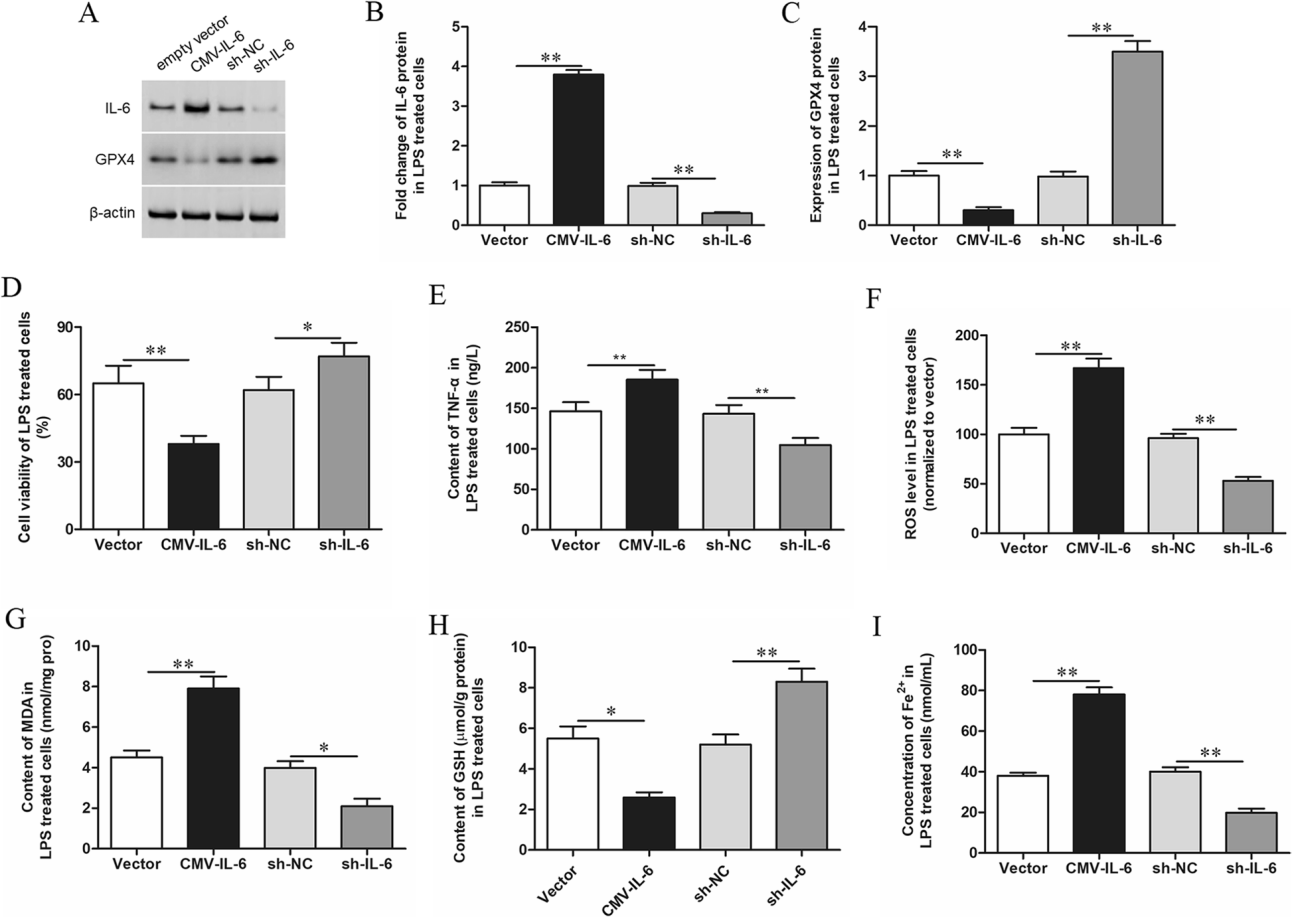
Effects of IL-6 on ferroptosis of LPS treated GMECs. LPS treated GMECs were transfected with CMV-IL-6 expression vector or IL-6 shRNA. **A**, **B** The overexpression and knockdown efficiency were detected by Western blotting and the grayscale was analyzed by using ImageJ software. **A**, **C** The expression level of GPX4 was detected by Western blotting and the grayscale was analyzed by using ImageJ software. **D** Cell viability was detected by CCK-8 assay. **E** The secretion of TNF-α was detected by ELISA. **F** The content of ROS was analyzed with DCFDA-cell ROS detection kit. **G** MDA content was detected by MDA assay kit. **H** The GSH levels were detected by GSH assay kit. **I** The concentration of Fe^2+^ was measured by using an iron assay kit

After overexpression or knockdown of IL-6, the inflammatory response, oxidative stress level and the expression of ferroptosis markers in LPS treated GMECs were detected. And it was found that overexpression of IL-6 down-regulated the expression of GPX4 (Fig. 2A-C), promoted the secretion of inflammatory factors (Fig. 2E), up-regulated the oxidative stress level (Fig. 2F-H), and promoted Fe^2+^ accumulation (Fig. 2I). That is to say, overexpression of IL-6 promoted the ferroptosis of GMECs treated with LPS and down-regulated cell viability (Fig. 2D). Interfering with IL-6 played the opposite role by inhibiting inflammatory response and down-regulating the level of oxidative stress, and inhibited the occurrence of ferroptosis (Fig. 2).

### USP14 was up-regulated in LPS-treated GMECs, and overexpression of USP14 promoted IL-6 expression

**Fig. 3:**
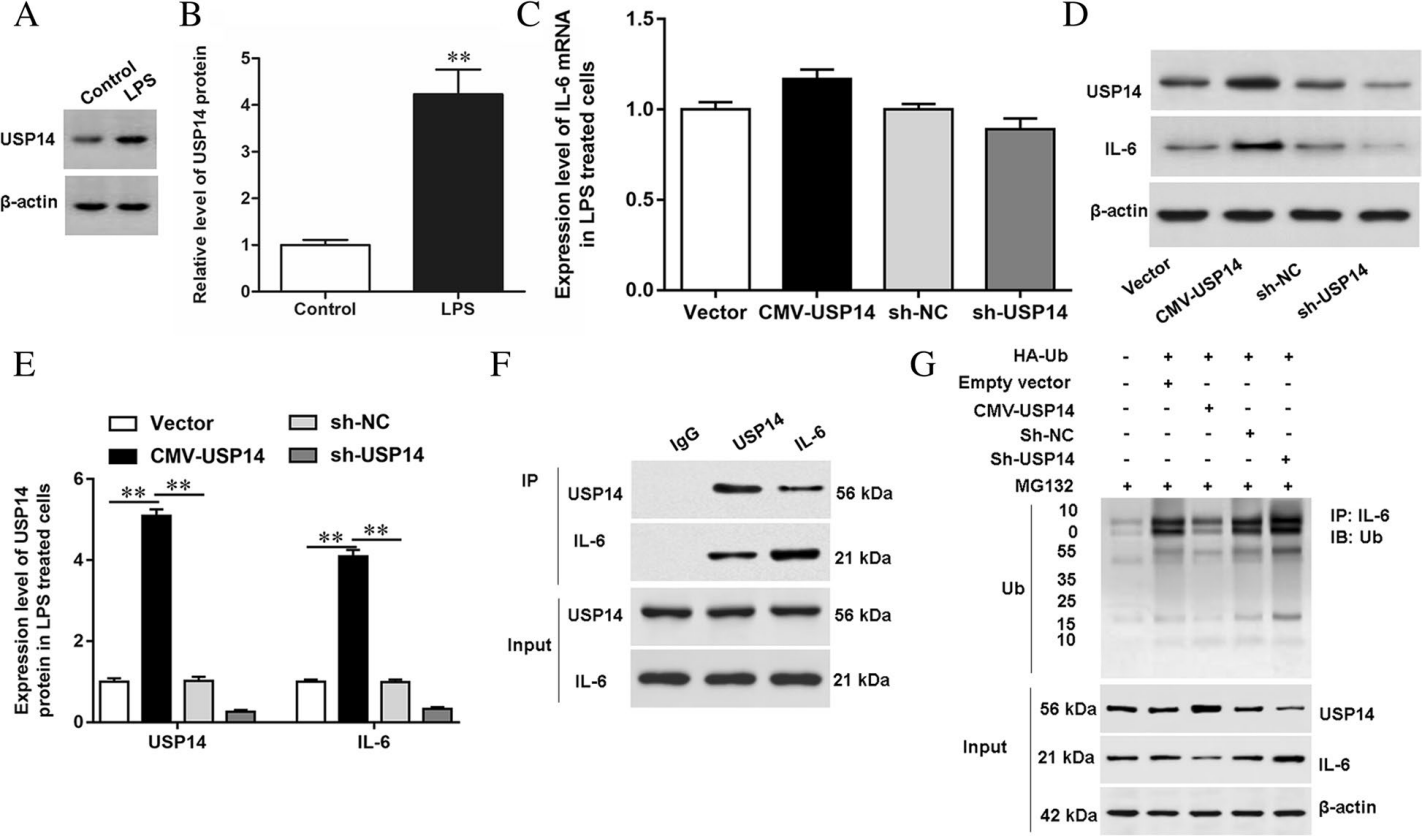
Effects of USP14 on IL-6 expression. **A**, **B** GMECs were treated with LPS, the expression level of USP14 protein was detected by Western blotting and the grayscale was analyzed by using ImageJ software. **C-E** LPS treated GMECs were transfected with CMV-USP14 expression vector or USP14 shRNA, the mRNA and protein expression levels of USP14 and IL-6 were detected by RT-qPCR and Western blotting, respectively. **F** The interaction between USP14 and IL-6 was verified by co-IP assay. **G** The effect of USP14 on the expression of IL-6 protein was detected by ubiquitin experiment

The expression level of USP14 protein was up-regulated in GMECs treated with LPS (Fig. 3A, B). After overexpression or knockdown of USP14, the mRNA and protein expression levels of IL-6 were detected by RT-qPCR and Western blotting, respectively. And it was found that interfering the expression of USP14 significantly decreased the expression of IL-6 protein, but had little effect on the mRNA expression levels of IL-6 (Fig. 3C-E). Subsequently, the interaction between USP14 and IL-6 was further verified by co-IP assay (Fig. 3F). The experimental results showed that there was a mutual combination between USP14 and IL-6 (Fig. 3F and Supplemental Figure 1). Next, the effect of USP14 on the ubiquitination level of IL-6 was tested, and the results showed that overexpression of USP14 down-regulated the ubiquitination level of IL-6, and knocking down of USP14 significantly increased the ubiquitination level of IL-6, which indicated that USP14 bound to IL-6 and enhanced the stability of IL-6 protein through deubiquitination (Fig. 3G).

### Overexpression of IL-6 reversed the effect of USP14 shRNA on ferroptosis in LPS-treated GMECs

**Fig. 4:**
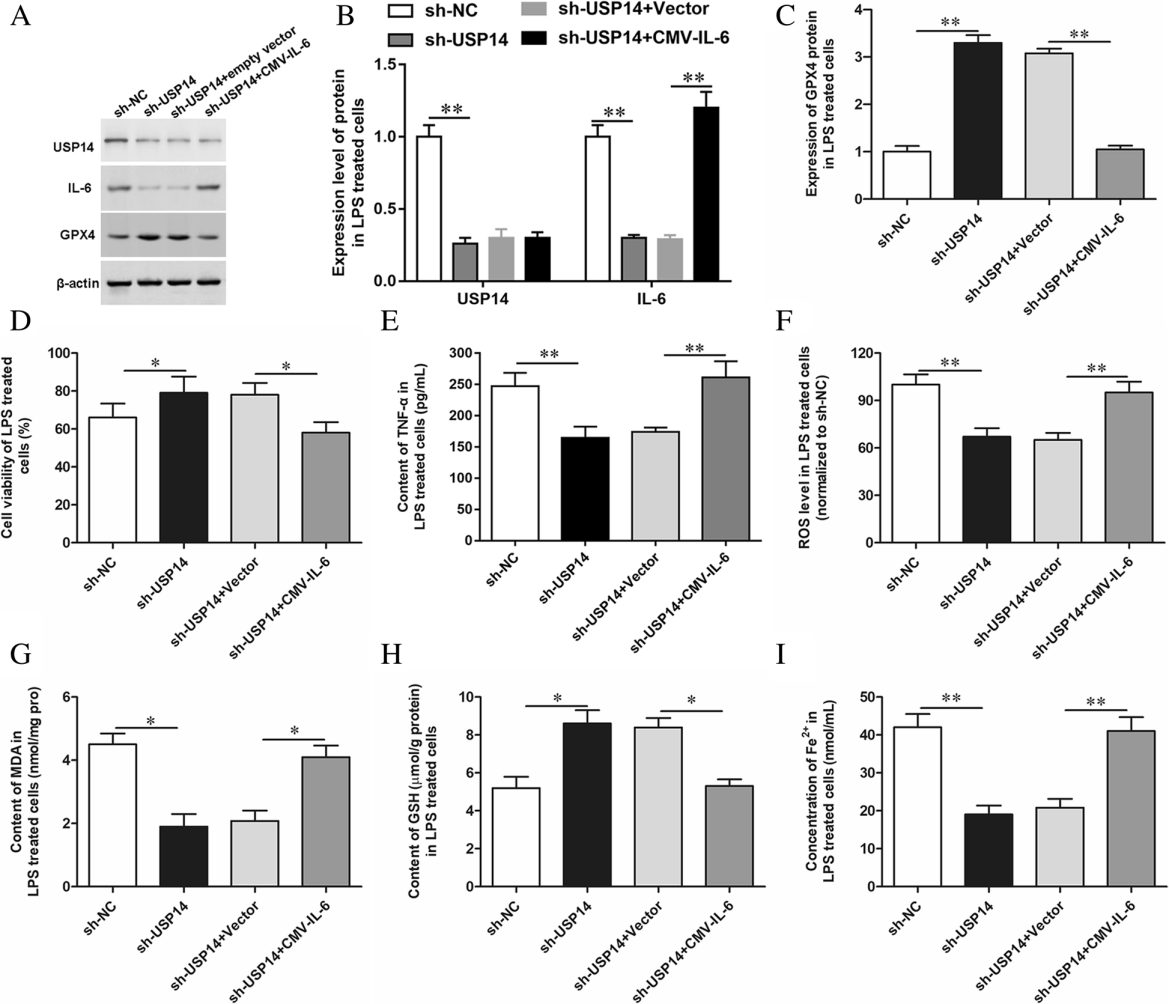
Overexpression of IL-6 reversed the effect of USP14 shRNA on ferroptosis of LPS-treated GMECs. LPS-treated GMECs were transfected with USP14 shRNA alone or together with CMV-IL-6. **A-C** The expression of USP14, IL-6, and GPX4 protein was detected by Western blotting and the grayscale was analyzed by using ImageJ software. **D** Cell viability was detected by CCK-8 assay. **E** The secretion of TNF-α was detected by ELISA. **F** The content of ROS was analyzed with DCFDA-cell ROS detection kit. **G** MDA content was detected by MDA assay kit. **H** The GSH levels were detected by GSH assay kit. **I** The concentration of Fe^2+^ was measured by using an iron assay kit

To further explore the regulatory mechanism of USP14, we transfected LPS-induced GMECs with USP14 shRNA and showed that silencing USP14 significantly reduced LPS-induced secretion levels of the inflammatory cytokines IL-6, TNF-α, IL-1β and IL-12 (Supplemental Figure 2). Next, USP14 shRNA and CMV-IL-6 were co-transfected into LPS-treated GMECs, and the levels of cell inflammation response, oxidative stress level and the expression of ferroptosis markers were further tested. It was found that overexpression of IL-6 could reverse the inhibition effect of USP14 shRNA on ferroptosis (Fig. 4).

### Overexpression of IL-6 reversed the effect of USP14 shRNA on the NRF2 signaling pathway in LPS-treated GMECs

**Fig. 5:**
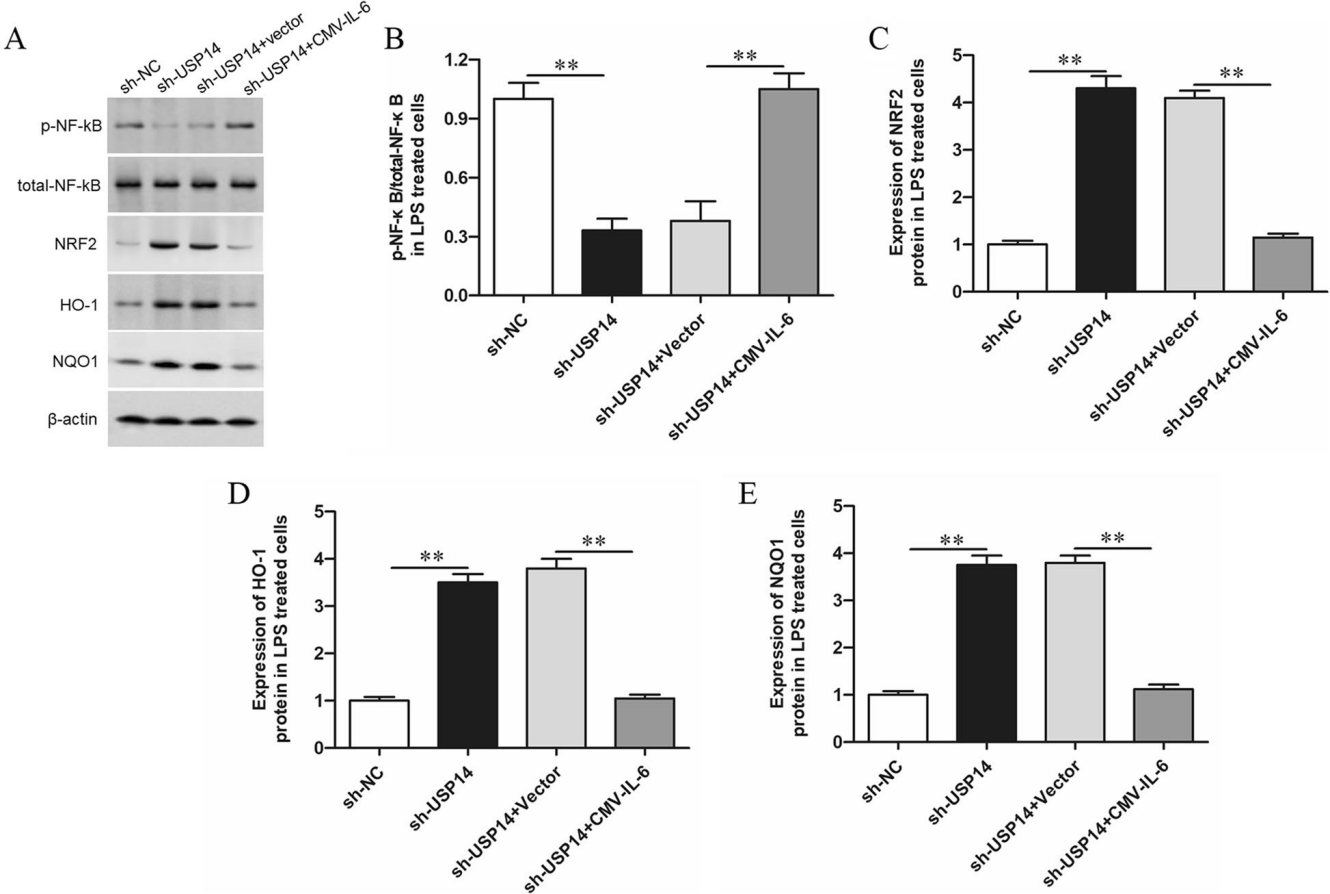
Overexpression of IL-6 reversed the effect of USP14 shRNA on the NRF2 signaling pathway in LPS-treated GMECs. LPS-treated GMECs were transfected with USP14 shRNA alone or together with CMV-IL-6, the protein expression levels of the NF-κB pathway and the NRF signaling pathway were detected by Western blotting and the grayscale was analyzed by using ImageJ software

USP14 shRNA and CMV-IL-6 were co-transfected into LPS-treated GMECs, and the protein expression levels of the NRF signaling pathway and its downstream genes were detected by Western blotting. It was found that knocking down USP14 activated the NRF2 signaling pathway and inhibited the activity of NF-κB pathway, overexpression of IL-6 could reverse the activation of USP14 shRNA on the NRF2 signal pathway (Fig. 5).

### Inhibition of NRF2 signaling pathway could reverse the effect of IL-6 shRNA on ferroptosis of LPS-treated GMECs

**Fig. 6:**
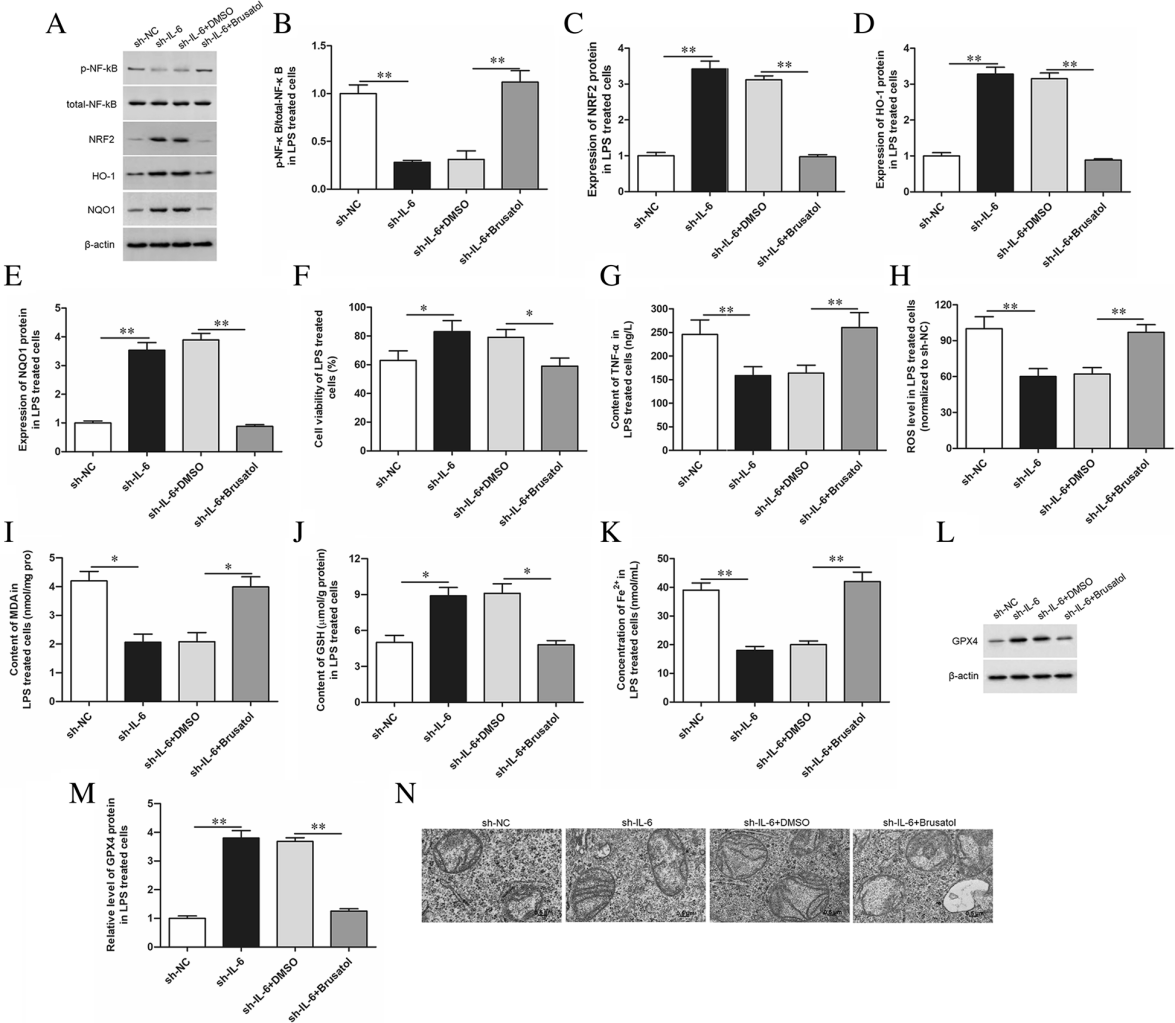
Inhibition of NRF2 signaling pathway reversed the effect of IL-6 shRNA on ferroptosis of LPS-treated GMECs. LPS-treated GMECs were transfected with IL-6 shRNA alone or together treated with the NRF2 signaling protein inhibitor Brusatol. **A-E** The expression of NRF2 and its downstream pathway proteins were detected by Western blotting and the grayscale was analyzed by using ImageJ software. **F** Cell viability was detected by CCK-8 assay. **G** The secretion of TNF-α was detected by ELISA. **H** The content of ROS was analyzed with DCFDA-cell ROS detection kit. **I** MDA content was detected by MDA assay kit. **J** The GSH levels were detected by GSH assay kit. **K** The concentration of Fe^2+^ was measured by using an iron assay kit. **L**, **M** The expression level of GPX4 was detected by Western blotting and the grayscale was analyzed by using ImageJ software. **N** Represented images of transmission electron microscopy

After knocking down the expression of IL-6, the cells were treated with NRF2 signaling protein inhibitor Brusatol (0.3 μmol/L; #SML1868, Sigma-Aldrich). And the expression of NRF2 and its downstream pathway proteins were detected by Western blotting (Fig. 6A-E). The inflammatory factor (Fig. 6G), oxidative stress level (Fig. 6H-J) and the expression of ferroptosis markers (Fig. 6K-N) were detected by specific kits, respectively. The results showed that Brusatol could reverse the inhibitory effects of IL-6 shRNA on the secretion of cell inflammatory factors (Fig. 6G), oxidative stress levels (Fig. 6H-J) and ferroptosis (Fig. 6K-M). And the electron microscopy showed that mitochondrial membrane densities were condensed, and mitochondrial inner membrane folds were reduction in GMECs treated with LPS. Knockdown of IL-6 alleviated the above phenomenon, and Brusatol treatment reversed the alleviating effect (Fig. 6N).

## Discussions

Mastitis is one of the diseases that are common in the development of the dairy industry and causes major economic losses. The occurrence and development of mastitis is a process of interaction between pathogenic microorganisms and breast defense system, which is determined by the pathogenicity of the pathogen and the strength of breast defense function. Mammary epithelial cells are the main parenchymal cells in the breast, the dysfunction of which is a main pathological mechanism of mastitis. Recently, it was revealed that ferroptosis, a new type of programmed cell death, which is iron dependent and different from apoptosis and cell necrosis and pyroptosis, was involved in the dysfunction of mammary epithelial cells. Inhibition of ferroptosis by biological or chemical means has been demonstrated to improved functions of mammary epithelial cells, including cell viability, oxidative stress and inflammation. In this study, we also found that LPS treatment increased oxidative stress, inflammation and ferroptosis of mammary epithelial cells, and inhibition of ferroptosis by USP14 knockdown or IL-6 knockdown could alleviate dysfunction of mammary epithelial cells [22, 23].

Cells with ferroptosis ultimately die due to mitochondrial dysfunction and lipid peroxidation toxicity [24]. In the biochemical process, the depletion of intracellular GSH, the decrease of GPX4 activity, the failure of lipid peroxides to be metabolized by GPX4 catalyzed reduction reaction, the accumulation of Fe^2+^ and a large amount of ROS produced by lipid peroxidation leading to the occurrence of ferroptosis [25]. Cell survival rate, the activities of GPX, SOD, CAT and the content of MDA reflect the ability of free radical scavenging and the degree of lipid peroxidation damage, which can be used as important indicators to determine whether cells are experiencing oxidative stress [26–28]. The secretion of cytokines interleukin (IL) and tumor necrosis factor-α (TNF-α) can also reflect the degree of oxidative damage of cells to a certain extent, and can be used as a reference index for judging the degree of cell damage [29, 30]. But TNF-α of activation mediates necroptosis under caspase-deficient conditions, does not mediate ferroptosis of cells [31]. In this study, we found that IL-6 protein expression was up-regulated in GMECs treated with LPS. The expression of oxidative stress and ferroptosis markers were further detected, and it was found that Fe^2+^ content was increased, and oxidative stress levels were up-regulated, which was relieved after Fer-1 treatment. The above changes indicated that LPS stimulated ferroptosis of GMECs, and knocking down of IL-6 or USP14 in LPS-treated GMECs inhibited ferroptosis induced by LPS.

IL-6 is a famous pro-inflammatory factor involved in a large number of acute and chronic inflammatory diseases. As a major pro-inflammatory cytokine induced by LPS, IL-6 was recently reported to play an important role in ferroptosis. For example, in mice with hemochromatosis, IL-6/hepcidin signaling was activated and contributed to ferroptosis of mouse hepatocytes, which could be suppressed by the anti-inflammation drug auranofin [32]. IL-6 and its receptor were aberrantly expressed in injured cartilage tissues of patients with intervertebral disc degeneration, and miR-10a-5p suppressed IL-6 expression and inhibited IL-6 mediated cartilage cell oxidative stress and ferroptosis [20]. Moreover, IL-6 was reported to regulate ferroptosis of epithelial cells in several organs [33, 34]. In our study, we found that IL-6 could induce ferroptosis of mammary epithelial cells, and inhibition of IL-6 by USP14 knockdown suppressed ferroptosis, suggesting a novel contribution manner of IL-6 in mastitis.

The ubiquitin–proteasome system mediates the degradation of most proteins in eukaryotic cells, and has various biological functions, such as regulation of inflammation, cell proliferation, signal transduction, transcription regulation, and apoptosis. USP14 is an important deubiquitinating enzyme. It is involved in the process of ubiquitin-mediated proteasome degradation of protein. It has been widely studied in the research of tumors, neurological diseases and aging, but its role in inflammatory response has been rarely reported. In breast cancer cells, knocking down USP14 reduced the protein expression of CDK1 by increasing the ubiquitination level, thereby arresting the cell cycle in the G2/M phase, and significantly reducing cell proliferation [35]. In this study, we found that the expression level of USP14 was up-regulated in LPS-treated GMECs, and USP14 up-regulated the expression of IL-6 protein by deubiquitinating IL-6. Knockdown of USP14 inhibited the occurrence of LPS-induced ferroptosis. And overexpression of IL-6 could reverse the inhibitory effect of knockdown of USP14 on LPS-induced ferroptosis.

NRF2 is an important antioxidant and anti-inflammatory regulator. It combines with downstream anti-oxidation response element (ARE) to control the transcription of GPX4, heme oxygenase-1 (HO-1), NADPH quinone oxidoreductase 1 (NQO1) and other genes [36]. However, the way NRF2 regulates inflammatory factors is controversial. On the one hand, NRF2 can inhibit the degradation of IκB-α proteasome and the activation of NF-κB signaling pathway, preventing NRF2 translocation into the nucleus to promote the expression of downstream pro-inflammatory genes IL-6, etc [37]. On the other hand, there are reports shown that NRF2 induces the expression of pro-inflammatory genes (including IL-6, IL-1α, etc.) through antioxidant response elements within pro-inflammatory gene promoters [38, 39]. In addition, NRF2 is closely related to multiple ferroptosis-related genes, such as antioxidant and iron metabolism, and is currently considered to be an important negative regulator of ferroptosis. NRF2 reduces toxic iron ions by regulating transferrin receptor (TFR1), FPN and FTH1, thereby enhancing the storage capacity of iron [40, 41]. On the other hand, GSH, GPX4 and xCT are regulated to increase the reduction capacity and inhibit lipid peroxidation [42, 43]. In the study, we found that knockdown of USP14 or IL-6 could activate the NRF signaling pathway, and NRF2 pathway inhibitor Brusatol reversed the inhibitory effect of knockdown of IL-6 on LPS-induced ferroptosis.

In conclusion, IL-6 protein expression can be regulated by deubiquitylation of USP14, and further influence the occurrence of LPS-induced ferroptosis through the NRF2 signaling pathway.

## Supplementary Information

**Additional file 1.**

## Data Availability

The datasets used during the present study are available from the corresponding author on reasonable request.

## Abbreviations

IL-6: Interleukin-6
GMECs: Goat mammary epithelial cells
LPS: Lipopolysaccharide
Fer-1: Ferrostatin-1
MECs: Mammary epithelial cells
E. coli: Escherichia coli
TLR-4: Toll-like receptor
FBS: Fetal bovine serum
EGF-1: Epidermal growth factor 1
ROS: Reactive oxygen species
GSH: Glutathione
MDA: Malondialdehyde
TBA: Thibabituric acid
Co-IP: Co-immunoprecipitation
TEM: Transmission electron microscope
SDS-PAGE: Sodium dodecyl sulfate polyacrylamide gel electrophoresis
ARE: Anti-oxidation response element
HO-1: Heme oxygenase-1
NQO1: NADPH quinone oxidoreductase 1
TFR1: Transferrin receptor
