# Supplementary material for: Inhibition of USP14 suppresses ferroptosis and inflammation in LPS-induced goat mammary epithelial cells through ubiquitylating the IL-6 protein
Source: Hereditas. 2022 May 12;159:21. doi: 10.1186/s41065-022-00235-y (PMC9102600; doi:10.1186/s41065-022-00235-y)
Supplement: Supplementary file 1 — Additional file 1. [file 41065_2022_235_MOESM1_ESM.docx]

**Supplementary figure legends**


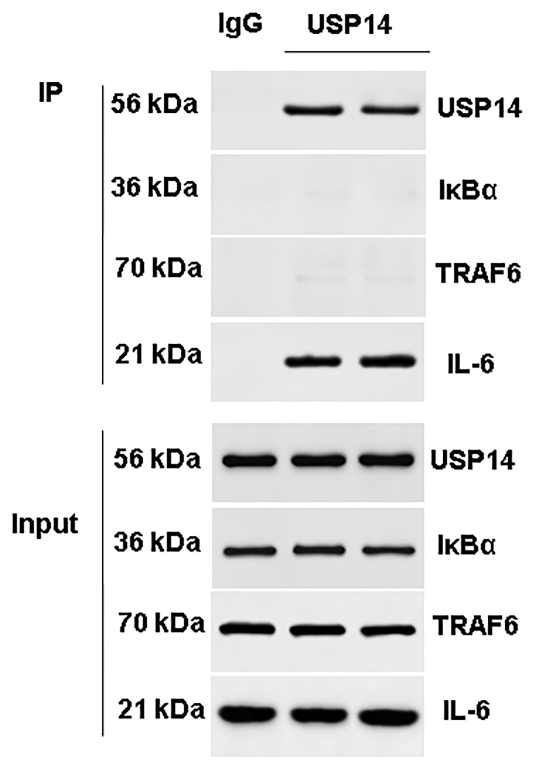


**Supplementary figure 1. USP14 interacted with IL-6.** The interaction between USP14 and IkBα, TRAF6, and IL-6, respectively, was verified in goat mammary epithelial cells with co-IP assay.


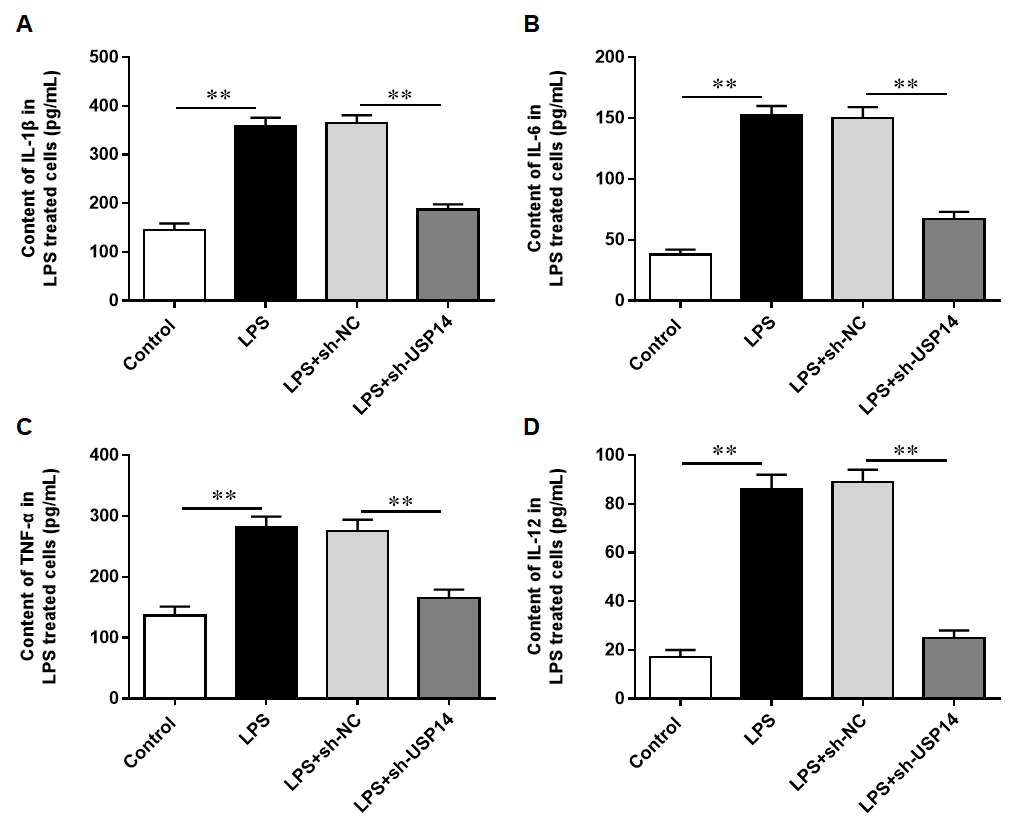


**Supplementary figure 2. Effect of silencing USP14 on the expression of inflammatory factors.** LPS treated GMECs were transfected with USP14 shRNA, and the secretion of IL-1β (A), IL-6 (B), TNF-α (C), and IL-12 (D) was detected by ELISA.
